# Composite Sampling Approaches for *Bacillus anthracis* Surrogate Extracted from Soil

**DOI:** 10.1371/journal.pone.0145799

**Published:** 2015-12-29

**Authors:** Brian France, William Bell, Emily Chang, Trudy Scholten

**Affiliations:** TDA Research Inc., Wheat Ridge, Colorado, United States of America; ContraFect Corporation, UNITED STATES

## Abstract

Any release of anthrax spores in the U.S. would require action to decontaminate the site and restore its use and operations as rapidly as possible. The remediation activity would require environmental sampling, both initially to determine the extent of contamination (hazard mapping) and post-decon to determine that the site is free of contamination (clearance sampling). Whether the spore contamination is within a building or outdoors, collecting and analyzing what could be thousands of samples can become the factor that limits the pace of restoring operations. To address this sampling and analysis bottleneck and decrease the time needed to recover from an anthrax contamination event, this study investigates the use of composite sampling. Pooling or compositing of samples is an established technique to reduce the number of analyses required, and its use for anthrax spore sampling has recently been investigated. However, use of composite sampling in an anthrax spore remediation event will require well-documented and accepted methods. In particular, previous composite sampling studies have focused on sampling from hard surfaces; data on soil sampling are required to extend the procedure to outdoor use. Further, we must consider whether combining liquid samples, thus increasing the volume, lowers the sensitivity of detection and produces false negatives. In this study, methods to composite bacterial spore samples from soil are demonstrated. *B*. *subtilis* spore suspensions were used as a surrogate for anthrax spores. Two soils (Arizona Test Dust and sterilized potting soil) were contaminated and spore recovery with composites was shown to match individual sample performance. Results show that dilution can be overcome by concentrating bacterial spores using standard filtration methods. This study shows that composite sampling can be a viable method of pooling samples to reduce the number of analysis that must be performed during anthrax spore remediation.

## Introduction

If an airport or seaport is shut down by biological agent contamination, the economic loss for each missed day would be enormous; it is absolutely essential to restore operations as rapidly as possible. Improved decon methods such as an electrochemical decon system (eClO_2_) produces 100% kill of anthrax spores in less than one minute [1]. To demonstrate that a large, complex area is clear requires taking and analyzing thousands of samples. In a crisis, decontamination equipment could potentially be assembled to treat an entire area in a matter of days, but using current sampling methods, many months to years would still be required to analyze samples and re-treat areas that show surviving spores.

The remediation activity would require environmental sampling, both initially to determine the extent of contamination (hazard mapping) and post-decon to determine that the site is free of contamination (clearance sampling). Whether the spore contamination is within a building or outdoors, collecting and analyzing what could be thousands of samples can become the factor that limits the pace of restoring operations. Consider anthrax spore contamination of a large U.S. airport with an area of 140 km^2^ (Denver International Airport), estimated to consist of 20% asphalt, 10% buildings and 70% open fields. If it is assumed that one sample is taken for every 5000m^2^ (roughly a football field) on the open ground, every 500m^2^ on asphalt, and every 100m^2^ on buildings. Using these sampling densities, there will be 84,348 samples to evaluate. Based on traditional plating techniques a single lab can do 40 samples in 48 hours, and so would require 12 years to complete these samples. To complete the job in two weeks would require 302 labs. Using advanced detection methods (RV-PCR) with a sample rate of 150 samples every 48 hours, it would take 3 years for one laboratory to complete the analysis; to get it done in two weeks would require 81 laboratories [2].

To address this sampling and analysis bottleneck, composite sampling was investigated to significantly decrease the number of samples that must be analyzed, thereby speeding the recovery process. In composite sampling, multiple samples are combined into a composite sample or pool, which is tested for contamination (live spores in this case) [3]. If the pool is clear, then the entire group has no contamination. If the pooled sample shows contamination, either the entire area can be re-treated, or the area can be sampled in detail to further asses were the contamination is located. In either case, this approach can reduce the number of analyses that must be run by an order of magnitude or more.

## Background

In cases where a large number of samples must be analyzed, with a strong majority producing the same result, it is possible to dramatically reduce the number of analyses by pooling or grouping samples. This approach was described in 1943 by Dorfman [4] who proposed testing blood samples for syphilis by pooling them into groups rather than testing each sample individually. If a pool tests positive, the individuals in that pool will be retested so that the infected individuals can be identified; if the pool is negative, then a large amount of time is saved because only one test has to be run, rather than testing all samples in the pool (10, 100 or whatever pool size is selected). This pooling or composite sampling procedure can greatly reduce the analysis time and costs with no loss in accuracy [5].

The standard procedure is to test the composite; if it tests positive, then re-test the individual samples to identify all of the positive samples. In a wide area biological agent restoration event we could follow this procedure if desirable, but to save valuable time, the more likely course is simply to re-treat the area covered by the group that tests positive until decontamination is achieved.

Pooled or composite sampling has been well documented in the literature; see the monograph by Patil et al. [6]. It is commonly used in drug discovery, and has been applied to environmental sampling, including Superfund sites that were analyzed for the presence of polychlorinated biphenyls (PCBs) [6]. Piepel et al. [7] describe the plan for a proposed test to release *B*. *atrophaeus* spores in a building at Idaho National Laboratory, using composite sampling approaches to reduce sample numbers. Lancaster [8] describes a RCRA facility investigation at Los Alamos National Laboratory; the contamination included radionuclides plus mercury and other inorganics in a site approximately 40 feet long and 15 feet wide. These authors identify both some concerns and some promising approaches for environmental analysis. Drielak [9] describes composite sampling for the forensic investigation of a CBRN event. Emanuel et al. [10] describe sampling procedures in the event of a biological attack. These references document the validity of composite sampling for such situations, but tend to focus on statistical analysis, and none addresses the wide-area decon challenge.

The benefits of composite sampling techniques are obvious: pooling two samples halves the number of samples that must be analyzed. Pooling 20 samples reduces the number of samples by a factor of 20, accelerating data acquisition. Compositing 20 samples can reduce the number of analyses that must be performed in the above (DIA) example to 4,217 samples. Using the new RV-PCR technique, four labs could accomplish the task of analyzing the samples for an anthrax spore contamination event within 14 days, a viable and acceptable timeline. A recent paper by EPA researchers consider methods for composite sampling of an anthrax spore surrogate form a non-porous surface using a single swab on multiple sampling surfaces [11]; however, additional work is required to fill data gaps and validate the method through field demonstrations and development for implementation in an anthrax spore remediation event.

Traditional sampling and analysis procedures have been identified, tested and validated by the CDC. These sampling method procedures are available on the CDC website [12]. Much effort has gone into validating these procedures, and they should continue to be utilized. In addition, these described compositing techniques do not alter the approved analysis methods used to detect or quantify viable spores present in the samples. While new methods to improve this analysis are being developed, the tried and true standard is to plate the sample suspension on a growth medium so that colonies can be counted and the number of viable spores in the sample calculated. Our approach is to take samples that have been collected using established sampling methods, composite them to reduce the number of laboratory samples, and analyze them using established procedures. This work demonstrates that compositing methods can work in combination with accepted sampling and analysis methods.

One of the challenges of developing a composite sampling approach is defining exactly how it is best used in the field under real world conditions. In other words, what is the best practice for compositing samples? Using the procedures described above, we will define two general methods: the single medium composite method and post-sample composite method. Further description of these techniques is provided below. It is also worth pointing out that a combination compositing approach using both of these methods would be possible.

In the single media compositing method, a single sample media is used to sample multiple locations. For example, a macrofoam swab has four sides and thus sampling surfaces. In this compositing method, a single side of the swab could be used to sample a single location. Four different sample locations would then be sampled with a single swab, each using a new side of the swab. The swab is then analyzed using standard ‘individual sample analysis’ methods as though it was a traditional single sample. The significant advantage of this technique is that the number of sampling kits required to be prepared prior to the sampling process is reduced by a factor of four. This can represent a significant reduction in the cost and labor needed to prepare large numbers of sampling kits, and also reduce the number of samples that must be tracked through the sample handling and analysis system. Another advantage is that no modifications in laboratory analysis procedures are required, as the single sample media is tested using standard procedures [13]. The EPA has expressed interest in this because the Bio-Response Operational Testing and Evaluation (BOTE) project showed that the time and effort for sampling media preparation was significant [14]. The disadvantage of this approach is that only 4 locations can be composited thus limiting the potential benefits (both time and analysis cost) that come with composites made from a large number of locations.

In the post-sample composite method, a single sample is used to sample a single location; multiple samples from various locations are combined after the sampling process. These samples can be combined just prior to laboratory analysis or in the field by placing all samples to be composited together for laboratory analysis. The advantage to this method is that it maximizes flexibility; numerous samples can be composited (from two samples to many) and multiple sampling media types can be composited. The disadvantage is that all samples have to be prepared as if all individual samples would have to be taken, however laboratory sample preparation and analysis time would be greatly reduced.

While testing has been performed using either the single media compositing or post-sample compositing methods, there is no reason that both of these techniques cannot be used simultaneously. As will be described below, both of these techniques have been independently verified. Using these sample techniques, single media compositing samples could be taken, providing the advantage of requiring fewer sample swabs to be prepared. The samples could be combined prior to analysis using the post-sample compositing techniques to allow the analysis of more than four samples at one time. Thus the compositing of two four-sided swabs would allow a single analysis of 8 sample locations. This type of technique would reduce the number of samples that had to be prepared by a factor of 4 and further reduce the number of samples that must be analyzed by a factor of 8. Significantly reducing the time and cost required to prepare samples, and the number of samples that must be analyzed in the laboratory.

The work described in this paper was undertaken to demonstrate the proof-of-concept of composite sampling as a tool that Federal On-Scene Coordinators or others responsible for directing a wide area biodecon operation can use during the recovery process. The methods described here, in conjunction with a good statistical sampling plan, such as the Visual Sample Plan (VSP) software tool, (developed and maintained by Pacific Northwest National Laboratory [15]) will be critical in a successful and timely recovery from an anthrax spore event.

## Materials and Methods

For these experiments commercially available biological indicator spore suspensions of *B*. *subtilis* (1.9x10^8^ CFU/ml, NAMSA, SBS-08) were used as a surrogate for the biological agent *B*. *anthracis*.

Arizona test dust (Powder Technology, Inc.) was steam autoclaved in 250 mg portions in 20 mL glass vials, placed on their sides to maximize soil surface area exposed to steam, at 250°F for 45 mins. Potting soil (Miracle Grow Moisture Control Potting Mix) was steam autoclaved in 250 mg portions, in 20mL glass vials placed on their sides to maximize soil surface area exposed to steam at 273°F for 2 hrs. One liter of Butterfield buffer (BBT) was prepared with 26.22 g potassium dihydrogen phosphate (Sigma-Aldrich), 7.78 g sodium carbonate (Sigma-Aldrich), 1 L distilled water (SpectraPure RO/DI) and 1 mL Tween 80 (Sigma-Aldrich). The buffer was sterilized by autoclaving at 250°F for 60mins. Tryptic soy broth (TSB) agar plates were prepared using 30 g. TSB, 15 g agar and 1 L distilled water. The TSB is autoclaved (Tuttnauer 2340M) then poured into sterile petri dishes.

### Proof-of-Concept Compositing

Spore suspensions were diluted in BBT and then recovered using filtration and cultured to determine the recovered population colony forming units (CFU). Each diluted spore suspension is filtered through a sterile filter assembly with a 0.2μm filter (VWR). The vial is rinsed three times with sterile BBT dilution solution and then filtered with the same filter assembly. The filter membrane is removed and placed in a sterile glass jar with lid, along with 4–6 glass beads (VWR), a magnetic stir bar (Sigma-Aldrich) and ten milliliters of BBT dilution solution. The vial is then sonicated (SPT UC-0609) for 5 minutes and then placed on a stir plate for at least 30 minutes, or until the filter is completely macerated. The resulting solution is diluted and plated on TSB plates to determine number of CFU on the filter assembly.

### Arizona Test Dust Compositing

Droplets of *B Subtilis* (0.1mL, 2.1*10^5^ CFU/0.1mL dilution) were added to sterilized dust (250mg) and then left undisturbed for 20mins. Afterwards 1.9mL of BBT buffer was added to the mixture (theo. 1.05*10^4^ CFU/0.1mL in the tube). The mixture was vigorously shaken by hand, then vortexed for 5 mins, before allowing the soil particles to settle. The somewhat cloudy supernatant was removed and transferred to a sterilized vial. Then 0.1mL of the supernatant was diluted with 9.9mL of buffer to form a 10X dilution, from which 0.1mL was plated on TSB agar plates.

Composite samplings with 4, 10 and 20 individual samples were carried out by gravity filtration of the supernatant from each individual sample through a 0.2 micron filter funnel (VWR) then rinsed with BBT buffer. The membrane was removed and digested with desired volume of buffer, from which 0.1mL was plated on TSB agar plates (as described above).

### Sterilized Potting Soil Compositing

Droplets of *B Subtilis* (0.1mL, 2.1*10^3^ CFU/0.1mL dilution) were added to sterilized soil (250mg) and then left undisturbed for 20mins. Afterwards 3.9mL of buffer was added to the mixture (theo. 52.5CFU/0.1mL in the tube). The mixture was vigorously shaken by hand, vortexed for 5 mins, and then left to “soak” for an additional 20-30mins. The dark brown to black supernatant was removed and transferred to a sterilized vial, and 0.1mL was plated on TSB agar plates.

Composite samplings with 4, 10 and 20 individual samples were carried out by gravity filtration of the supernatant recovered from individual samples through a 0.2 micron filter funnel, then rinsed with buffer. The membrane was removed and digested with desired volume of buffer, from which 0.1mL was plated on TSB agar plates (as described above).

### Controls

Blank controls were performed to verify the *B*. *subtilis* CFU counts that were place on each sample. The results of these control population counts were used to calculate the recovery efficiency from each sample, or are directly presented in the data results. All samples were plated in triplicate and the average population count is reported in terms of colony forming units (CFU).

## Statistical Box Plots

The statistical box plots used in the figures are presented so that the data and box plot are overlapped. The box represents 25 to 75% of the data spread, and the whiskers are set to extend to two standard deviations of the data.

## Statistical Analysis

Hypothesis testing was performed using one-sample t-tests, comparing a single data point with a larger population, and two-sample t-tests, comparing two sample data sets together. In most cases the null hypothesis states that the two-sample populations are equal or that the single sample result matches the comparative sample mean. The level of significance for all tests was set at 0.05. The Origin 9.1.0 software package was used to perform the analyses.

## Results and Discussion

### Proof-of-concept Compositing

The CDC has published sampling procedures, emergency response resources—surface sample procedures for *Bacillus anthracis* spores on smooth, non-porous surfaces—revised April 16, 2012 [5]. Based on those verified procedures, the macrofoam swab and cellulose sponge sampling use only a wetted swab or sponge for sampling. Once the material has been used to sample the contaminated surface it is placed in a screw cap container prior to analysis. In the laboratory, the samples are treated to remove the spores from the sampling media and 3 milliliters of spore suspension per sample remains [14]. From the three milliliters of spore suspension, samples are diluted and plated on growth media to determine the number of viable spores. Thus when compositing samples each sample represents a 3 milliliter volume and so combination of 100 samples means the volume to analyze is 300 milliliters.

The challenge for composite sampling is the dilution caused by the combination of these samples. For example, if only a single location is ‘hot’ and that sample is diluted with 99 other clean samples, instead of detecting the spores in 3 milliliters the same number of spores now have to be detected in 300. We tested whether concentrating the spores in suspension by filtration, prior to analysis, could overcome this dilution. A sterile 0.2 micron filter is used to filter the spore suspension and collect the individual spores. This filtration also allows any residual decontaminant or surfactants to be washed away. Once the filtration step has been completed the spores are recovered from the filter by washing and suspended in a small volume for analysis.

These tests used commercially available biological indicator spore suspensions (specifically, *B*. *subtilis*). TDA performed a population count on this spore suspension and determined that there were approximately 2.16 +/- 0.07 x10^8^ CFU per 0.1ml in the suspension.

**Fig 1:**
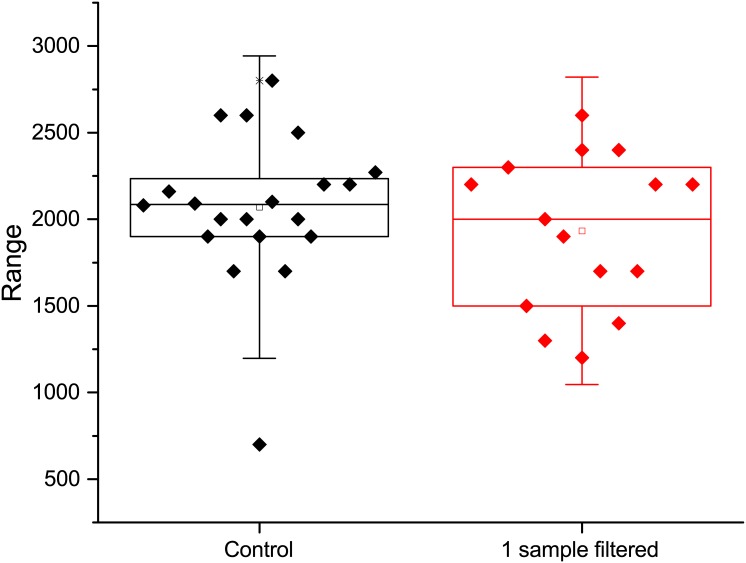
Box plot showing the data of the control non-filtered and filtered spore population counts.

Once this concentration of viable spores was identified, we assessed how the filtration process affects the recovery of spores. To accomplish this task we prepared three-milliliter sample solutions that each contained ~2000 CFU of spores. Some of these solutions were simply plated and counted, others were filtered, recovered and then plated and counted. This allows for the determination of any systematic deviation in the spore count caused by the filtration process. Based on the results of 15 growth plates from both the filtered and non-filtered control the results showed that on average 2070 +/- 486 CFU were recovered from the control and 1933 +/- 443 CFU from the filtered samples. The averages from these measurements are well within one standard deviation from each other and a two-sample t-test shows they are statistically equivalent (p = 0.36899; at the 0.05 level, the population mean is not significantly different from the test mean). A box plot of the data from the control and the filtered sample is shown in Fig 1.

Once it was established that the filtration process could successfully be accomplished and that there were no systematic losses of spores, we began to further dilute the samples as they would be during compositing to ensure that the spore could still be recovered. In other words, we took a fixed number of spores and diluted them to greater and greater amounts and then filtered them to ensure we could recover those spores. One concern is that as the volume of diluting solution became larger and larger, the spores may become lodged in the filter and become unrecoverable or that the filter might fail and some of the spores may be lost. We diluted a three milliliter spore suspension up to a volume of one liter, representing a compositing of 333 individual samples. This diluted solution was then filtered and the filter treated to recover the spores. A population count was then performed to determine the number of spores recovered. This count was then compared with the unfiltered control sample to determine if there was any deviation in the spore count: matched spore recovery is expected if the filtration process works correctly.

**Fig 2:**
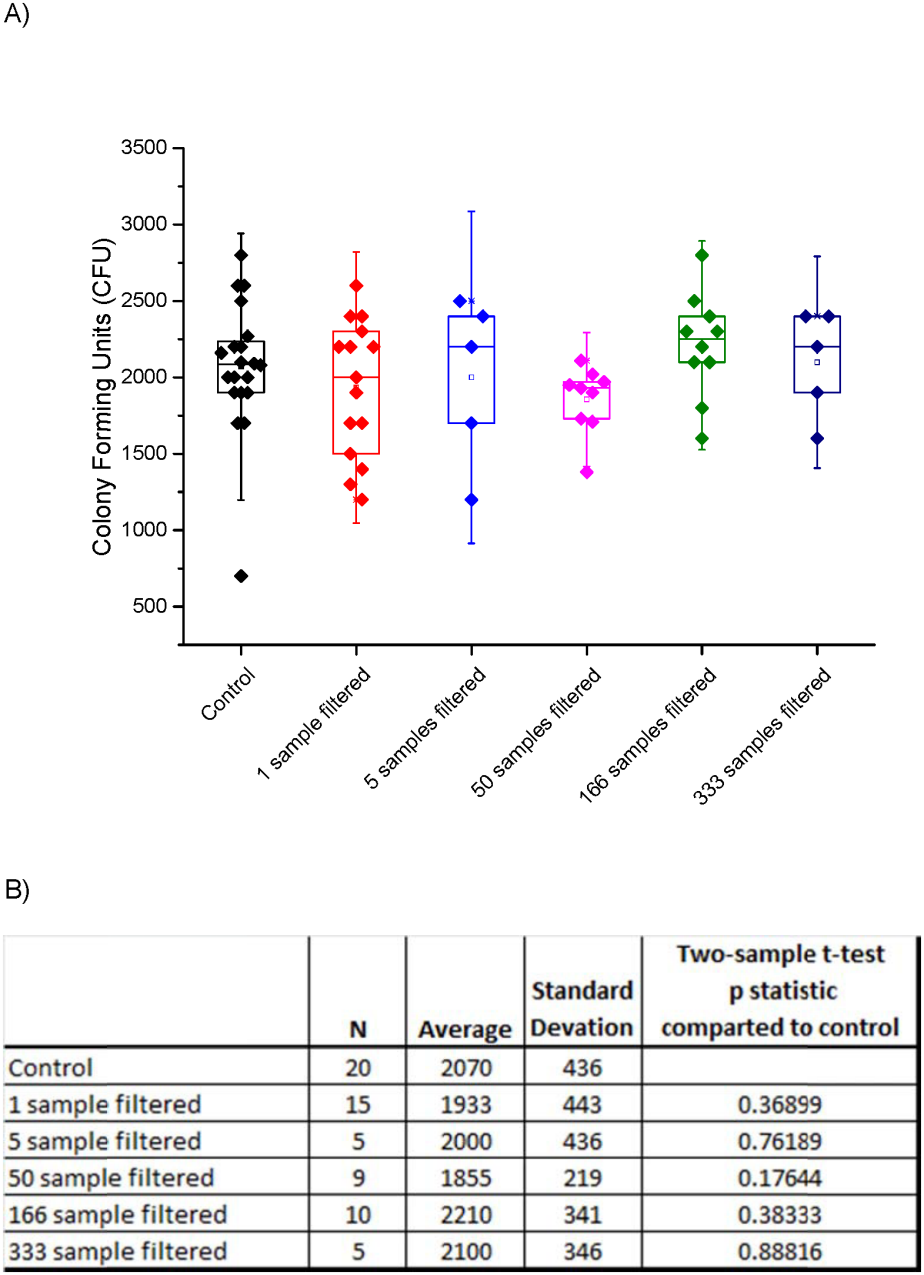
(A) Box plot of filtration spore recovery from samples diluted with volumes consistent with pooled sampling. Each starting sample had a volume of 3 ml. For example, the “333 sample” is a single 3-ml spore suspension diluted to 1 liter, filtered and then plated to determine the number of CFUs. The control is a non-filtered non-diluted spore count. (B) Table showing number of samples (N), average spore count, standard deviation and result of a two-sample t-test p statistic compared to the control samples. All samples were statistically matched to the non-filtered population control samples.

The results of this systematic spore suspension dilution and recovery experiments showed that even under a high dilution level, as would be expected after compositing numerous samples, the spores can be concentrated and recovered. A box plot of the colony counts in the control and the filtered sample is shown in Fig 2a. Composite samples comprising as few as 5 individual samples, and up to 333 individual samples have been filtered and the colony count determined in each case. Fig 2b provides the number of data points, average spore count, standard deviation and two-sample t-test p-statistic comparing each treated sample to the untreated population control. This data provides a proof of concept for the ability to combine samples, as would be required to analyze composite samples, and then concentrate them for analysis without losing the sensitivity to detect small numbers of spores.

These test data confirm that the dilution associated with pooling many samples does not lead to a decrease in sensitivity because the spores can be reconcentrated by filtration. Therefore, composite sampling can be particularly advantageous in detection of anthrax spores. There is an additional situation where concentration of samples by filtration may be desirable. Assume that the analytical method used has some limit of detection, or that there is a tradeoff between sensitivity and the time for analysis. Consider a situation in which the effective limit of detection is 10 spores. Now consider a collection of 300 samples, in which 20 are positive, each containing 5 spores. If each of the 300 samples is analyzed individually, none will be identified as positive. However, if all 300 samples are pooled, the resulting composite contains 100 spores, and will easily test positive. In this case, we have reduced the geographic resolution, but have obtained a correct result (avoiding false negatives) while lowering the time and cost of analysis.

#### Arizona Test Dust Compositing

**Fig 3:**
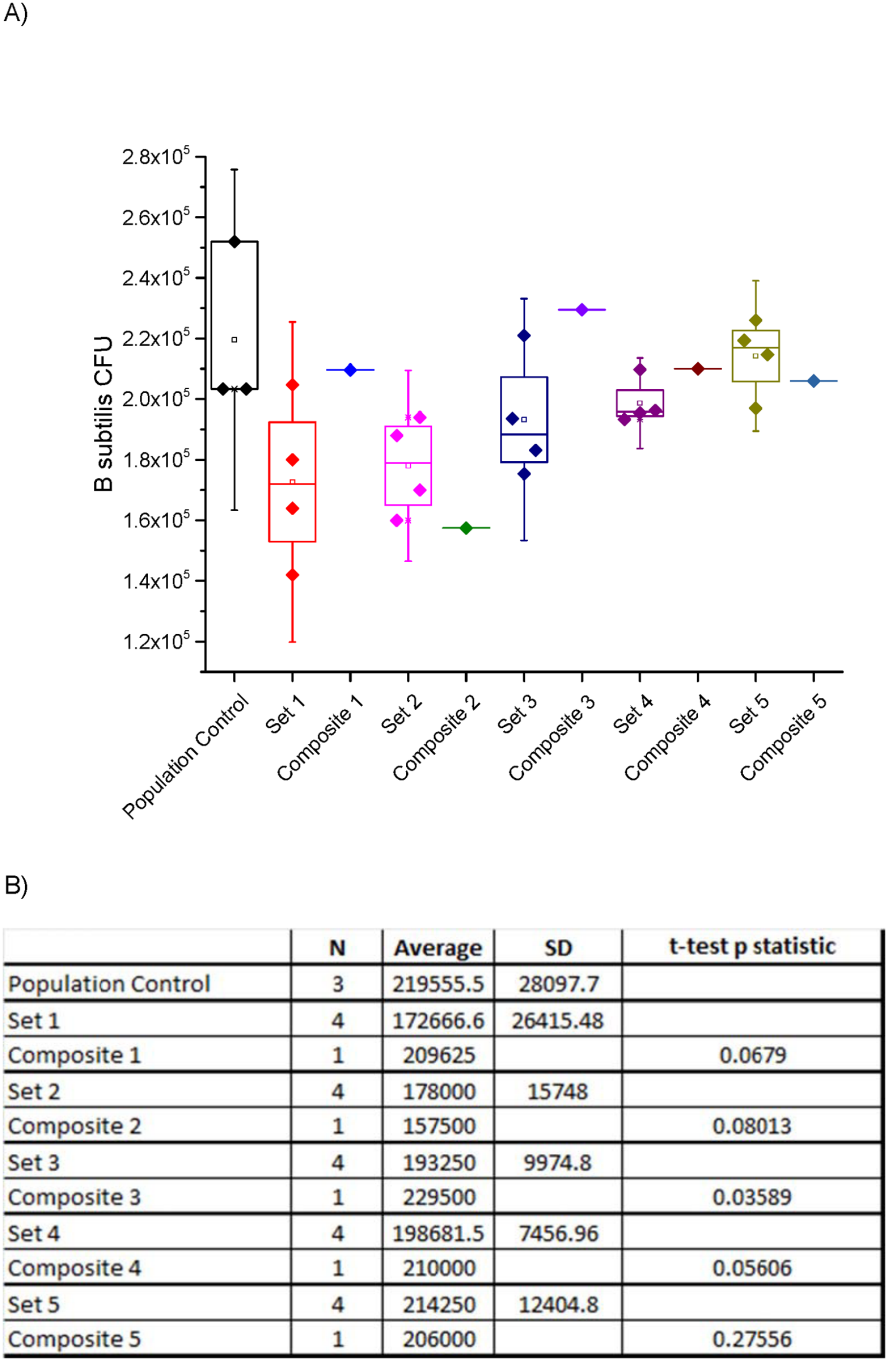
(A) Box plot of Arizona test dust samples, including the population control, individual analysis of each of the four samples in a composite (four data points), and their corresponding four-sample composite (single data point). (B) Table showing number of samples (N), average spore count, standard deviation and result of a one-sample t-test p-statistic comparing the individual sample sets and its corresponding composite pool sample.

In these tests, 2.2x10^5^ CFU of *B*. *subtilis* spores were added to each sample of Arixona Test Dust. Twenty individual samples were prepared; colony counts were determined for each individual sample, and also for composites of 4, 10 and 20 samples. Initially, four individual samples were combined, giving five sets of composite samples. Both the individual samples and the composites were analyzed. A box plot of the population control, individual samples and corresponding composites are shown below in Fig 3a. An average recovery from the individual samples of 86.7 +/- 12.7% was achieved. The experiments were performed by set order (1 through 5) which shows a general trend of improved recovery and reduced deviation.

The spore recovery from the five four-sample composites was 91.3 +/- 11.1%. Fig 3 shows there are no significant losses in the number of spores in the composited samples. One sample t-tests were used to compare the 5, four individual sample sets with their corresponding combined composite pooled sample. All samples sets and their corresponding composites were statistically matched, except set 3 (p = 0.03589, at the 0.05 level, the population mean is significantly different from the test mean). The composited sample for set 3 was higher than the individual samples; however, it was within two standard deviations of the individual samples that made up set 3. In addition, the composited sample for set 3 had nearly identical results (229,500 CFU) as the average for the spore population count (219,556 CFU) that was used to make these samples. Fig 3b shows the number of data points, average spore count, standard deviation and one-sample t-test p-statistic comparing the individual sample sets and its corresponding composite pool sample.

**Fig 4:**
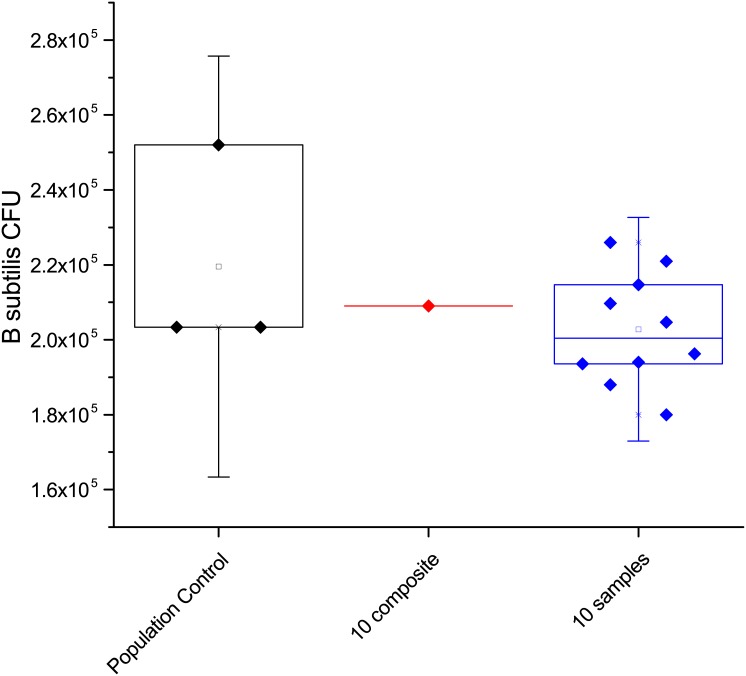
Spore recovery from Arizona test dust, from left to right are the data for population controls, 10-sample composite, and the 10 individual samples used in the 10-sample composite.

Using the twenty individual Arizona test dust recovery samples described above, the same methods that were used to composite four samples were used to prepare a composite of 10 individual samples. The results of these tests are shown in Fig 4, where the first column is the population control data, the results of the 10-sample composite followed by the 10 individual samples used to produce the composite. The spore recovery for the 10 sample composite is 99.5% of the spores that were added to the individual samples. A one-sample t-test of the composited and individual samples shows that they are matched with a p = 0.22062 (at the 0.05 level, the population mean is not significantly different from the test mean). This data shows that compositing of ten individual samples does not cause a loss in spores and nearly all the spores are recovered in a single analysis.

**Fig 5:**
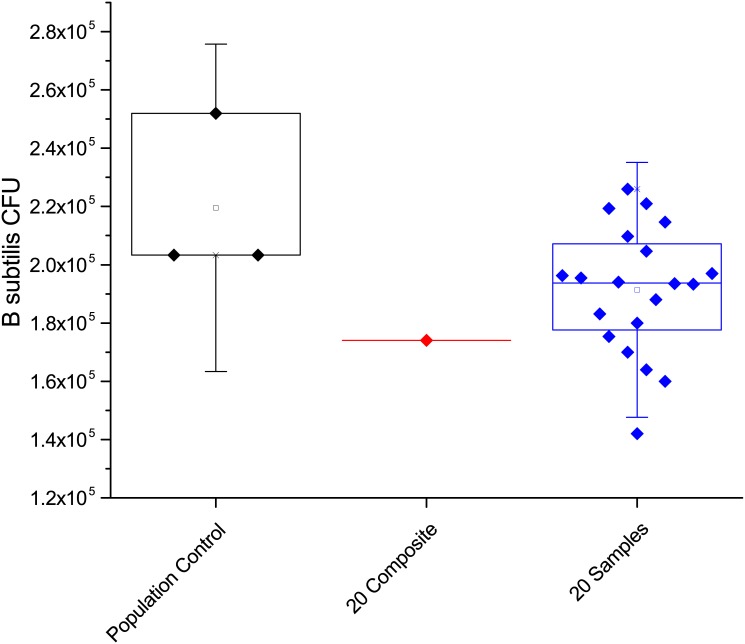
Spore recovery from Arizona test dust, from left to right are the data for the population controls, the 20-sample composite and then the results from the 20 individual samples that were composited.

Using the twenty individual Arizona test dust recovery samples described above, the same methods that were used to composite 4 and 10 samples were used to composite all twenty samples. The results of these tests are shown in Fig 5 where the first column is the population control data, the 20 sample composite followed by the results of the 20 individual samples used to produce the 20 sample composite. The spore recovery for the 20 sample composite is 82.9% of the spores that were added to the individual samples. This data shows that compositing twenty individual samples does not cause a significant loss in spores, and nearly all the spores are recovered in a single analysis. A two-sample t-test between the population control and 20 samples was performed and showed they were statistically equivalent (p = 0.217, at the 0.05 level, the population mean is not significantly different from the test mean). A one sample t-test between the 20 sample composite and the 20 samples used to make that composite was performed and showed that the composite was statistically low (p = 0.00214, at the 0.05 level, the population mean is significantly different from the test mean), however the 20 sample composite result is within one standard deviation of the results of the 20 individual samples.

Based on these spore recovery results on Arizona test dust we were able to show that the techniques and methods identified can be used to composite many samples into a single composite without loss of accuracy or dilution of spores in the sample. In theory, hundreds of samples could be composited using these techniques. Instead of continuing with larger numbers of individual samples we chose to move on to a more challenging soil substrate and reduce the number of spores used to contaminate each individual sample, thus increasing the challenge on our developed techniques.

#### Sterilized Potting Soil Compositing

In these tests we evaluated composite sampling of a more complicated soil matrix: steam-sterilized potting soil. Potting soil is much more complicated than Arizona Test Dust. Typical commercial potting soils contain peat, composted bark, sand, perlite and can include fertilizers and slow release nutrients in which plants can be grown. Potting soil is a next level of sophistication towards actual environmental sampling. To increase the challenge, in this test we also reduced the number of spores added to each individual sample, from 2.1x10^5^ CFU used on the Arizona test dust samples to under 2000 CFUs. The more complicated soil matrix and reduced number of spores did not require a change in the testing protocols. Despite this increased challenge, the compositing techniques that were demonstrated performed as desired.

**Fig 6:**
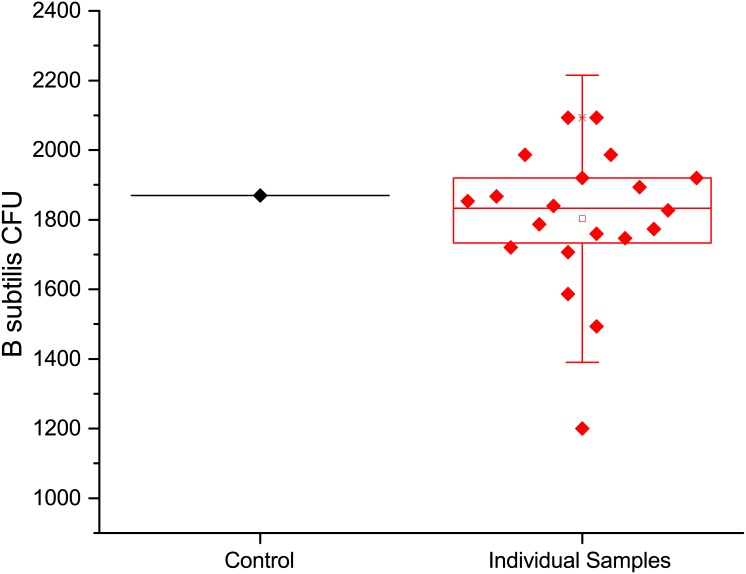
Population control and individual sample recovery from contaminated sterilized potting soil.

The same basic series of experiments that were performed on the Arizona test dust were also carried out on the sterilized potting soil. A single population control sample shows that 1870 CFU were being added to each individual sterilized soil sample. A box plot of the population control and results from the individual samples is shown in Fig 6. A one-sample t-test showed matched recovery of spores and the population control (p = 0.168, at the 0.05 level, the population mean is not significantly different from the test mean). The average spore recovery for the individual samples was 96.6 +/- 11.0%. The population control was within one standard deviation of the results of the 20 individual contaminated samples.

**Fig 7:**
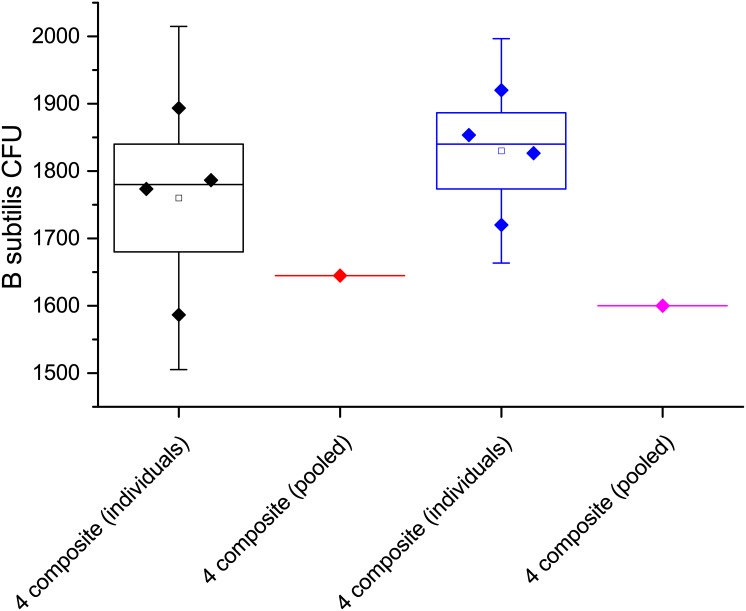
Two four-sample composites and their corresponding individual sample results. Samples were extracted from sterilized potting soil.

As with the Arizona test dust, we first used four-sample composites to test the compositing techniques. Two, four-sample composites were prepared; the colony counts for the individual samples and their corresponding composites are shown in Fig 7. The results on the left of Fig 7 show a composite that is within one standard deviation of the results of the four individual samples. A one-sample t-test showed matched recovery of the spores between the composite and individual samples (p = 0.168, at the 0.05 level, the population mean is not significantly different from the test mean). In the results shown on the right of Fig 7 the composite sample shows a result that is statistically low using a one-sample t-test (p = 0.01, at the 0.05 level, the population mean is significantly different from the test mean). The composite is outside two standard deviations of the individual samples, it is however within three standard deviations. This deviation was the largest observed in this study and while a majority of the spores were recovered (88%) additional testing was carried out with larger composites without seeing any significant loss in spores.

**Fig 8:**
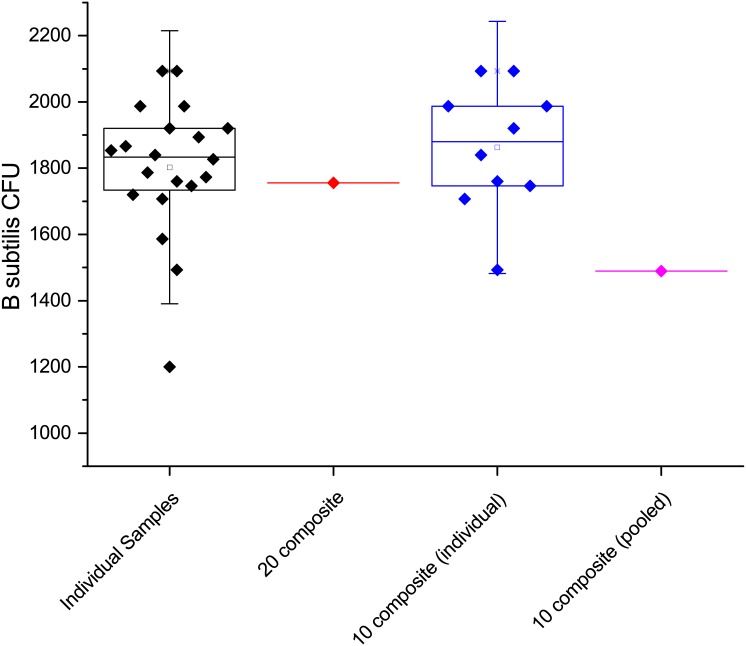
(left) 20 individual samples and their composite sample and (right) 10 individual samples and their composite.

As with the Arizona test dust samples, composites of 10 and 20 were also completed with the contaminated potting soil. The results of the 20 sample composite (both individual results and the composite result) are shown on the left side of Fig 8, while the right side shows the individual results and composite for the 10 sample analysis. The twenty-sample composite is within one standard deviation of the individual results and shows good agreement A one-sample t-test showed matched recovery of spores (p = 0.324, at the 0.05 level, the population mean is not significantly different from the test mean). The 10 sample composite is within two standard deviations of the individual results, a one-sample t-test suggested a statistical difference (p = 0.001, at the 0.05 level, the population mean is significantly different from the test mean).

**Fig 9:**
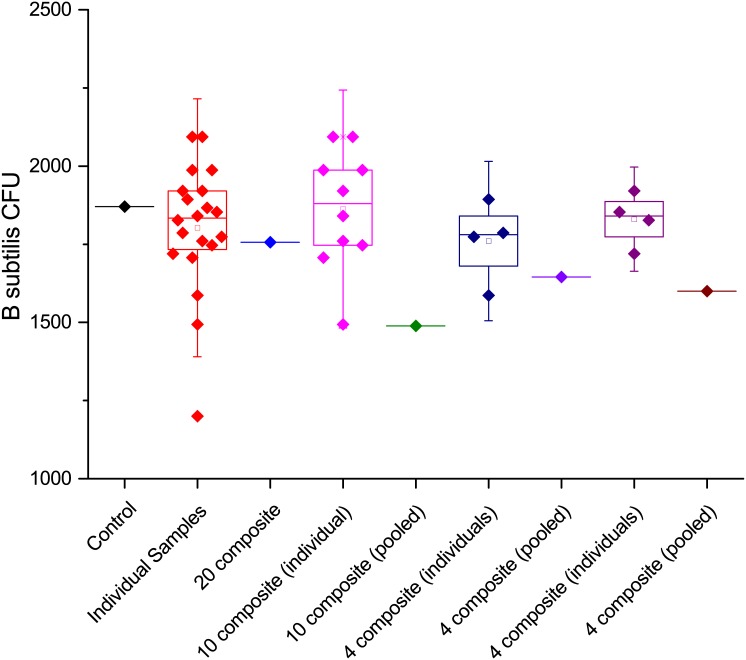
Summary data collected during spore recovery and compositing on sterilized potting soil.

A summary box plot of all of the data generated to demonstrate composite sampling and recovery of bacterial spores from sterilized potting soil is presented in Fig 9. Composites of up to twenty samples worked well with the techniques described above and the results appear well correlated with the individual samples used to make up the composite. The data suggest that composite samples from a larger number of individual samples could be effectively concentrated and analyzed.

## Conclusions

In the event of widespread anthrax spore contamination, the time required to obtain and analyze the samples needed to identify the location of contamination (hazard mapping) and verify decontamination (clearance sampling) constitute a bottleneck in the recovery process. In this effort a proof-of-concept study was performed to show that composite sampling of bacterial spore samples (surrogates for Anthrax) can be composited without spore loss due to dilution. A compositing methodology was described and demonstrated by recovering spores from contaminated soil substrates.

Preliminary testing was performed and demonstrated that spore suspensions can be successfully concentrated during composite sampling analysis to mitigate dilution issues. Composites as large as 333 samples were analyzed without loss in sensitivity. In addition, it was shown how composite sampling can actually improve the detection of low concentrations of anthrax spores over the analysis of individual samples.

Spore recovery from dirt samples were used to mimic real world environmental samples. The composite samples from the dirt recovered spore samples showed the laboratory techniques are viable and results represented those from the individual samples. No loss in sensitivity or loss of spores was seen or identified.
